# Shape-Memory-Reduced Graphene/Chitosan Cryogels for Non-Compressible Wounds

**DOI:** 10.3390/ijms24021389

**Published:** 2023-01-10

**Authors:** Hongyun Xuan, Qian Du, Ruimeng Li, Xiaoni Shen, Jiao Zhou, Biyun Li, Yan Jin, Huihua Yuan

**Affiliations:** School of Life Science, Nantong University, Nantong 226019, China

**Keywords:** shape memory, antibacterial, hemostatic, reduced graphene/chitosan cryogel, one-step method

## Abstract

In this study, an antibacterial and shape-memory chitosan cryogel with high blood absorption and fast recovery from non-compressible wounds was prepared using a one-step method. Herein, we prepared a shape-memory-reduced graphene/chitosan (rGO-CTS) cryogel using a one-step method with a frozen mixing solution of chitosan, citric acid, dopamine, and graphene oxide, before treating it with alkaline solutions. The alkaline solution not only promoted the double cross-linking of chitosan but also induced dopamine to form polydopamine-reducing graphene oxide. Scanning electron microscope (SEM) images showed that the rGO-CTS cryogel possessed a uniform porous network structure, attributing excellent water-induced shape-memory properties. Moreover, the rGO-CTS cryogel exhibited good mechanical properties, antibacterial activity, and biocompatibility. In mouse liver trauma models, the rGO-CTS cryogel showed good blood clotting and hemostatic capabilities. Therefore, this composite cryogel has great potential as a new hemostatic material for application to non-compressible wounds.

## 1. Introduction

Severe bleeding and infected wounds are key global issues that need to be addressed [1,2]. Uncontrolled hemorrhage caused by severe trauma is the main cause of shock and death. Meanwhile, the healing of infected bleeding wounds should not be underestimated as it may lead to serious problems, such as prolonged wound healing time, implant failure, and even death [3]. An ideal hemostatic agent must rapidly control massive hemorrhage, have antibacterial properties, and be biocompatible, lightweight, stable, easy-to-use, and inexpensive [4,5]. Current hemostatic agents, including cyanoacrylates, fibrin-based bandages, gelatin-based hemostatic agents, and zeolite-based QuickClot, have good hemostatic effects on superficial wounds but have limited effects on controlling deep incompressible bleeding. These hemostatic agents have drawbacks, such as poor adhesion to the wound surface, poor biocompatibility, and lack of effective antibacterial and healing effects [3,4]. 

To solve these issues, shape-memory materials have been developed that can rapidly stop bleeding and expand to fill and apply pressure to deep and non-compressible wounds. XStat ^TM^, composed of miniature sponges, can fill and apply pressure to deep and non-compressible wounds. However, owing to its low biocompatibility and non-degradable feature, it requires significant time to remove sponges from the wound. Subsequently, shape-memory cryogels with interconnected microporous structures have received extensive attention in biomedical applications. The special porous structure allows water to flow freely into or out of the cryogels. Thus, the cryogel shape can be controlled through compression to expel free water and change the cryogel shape and through absorbing water to restore its original shape. Hydrogels or cryogels usually show weak mechanical strength, which is due to their natural elements and microporous structure formed by a freeze-drying method used to remove ice crystals. To enhance their mechanical properties, Zhao et al. [4] introduced carbon nanotubes (CNTs) into quaternized chitosan (QCS) and fabricated nanocomposite cryogels for wound dressing. The cryogels can take seconds to recover their shape and have good mechanical and hemostatic properties. However, CNTs are potentially cytotoxic. To further improve the performance of porous cryogels, Guo’s group introduced polydopamine (PDA) into the QCS reaction system, where a multifunctional cryogel with tissue adhesion properties was prepared for rapid non-compressed wound hemostasis and healing [6]. Similarly, Cao and co-workers recently reported that a shape-memory cryogel based on QCS and bioactive glass could be used for rapid hemostasis and wound healing [7]. Therefore, the construction of biocompatible, high-strength, and easy-to-use shape-memory cryogel hemostatic agents has promising prospects for clinical application. 

Graphene oxide (GO), one of the important derivatives of graphene, has attracted much attention in recent years. It has been regarded as an effective reinforcement for chitosan films [8]. Furthermore, PDA-reduced graphene oxide (rGO) was chosen to strengthen chitosan hydrogel with a fast self-healing ability, and attributed to good adhesiveness and enhanced biodegradability [9]. We previously reported the preparation of improved strength chitosan (CTS) hydrogels using the green natural crosslinking agent citric acid [10]. Therefore, the CTS cryogel formed by the crosslinking of citric acid and PDA rGO will have excellent biomechanical and biochemical functions.

**Figure 1:**
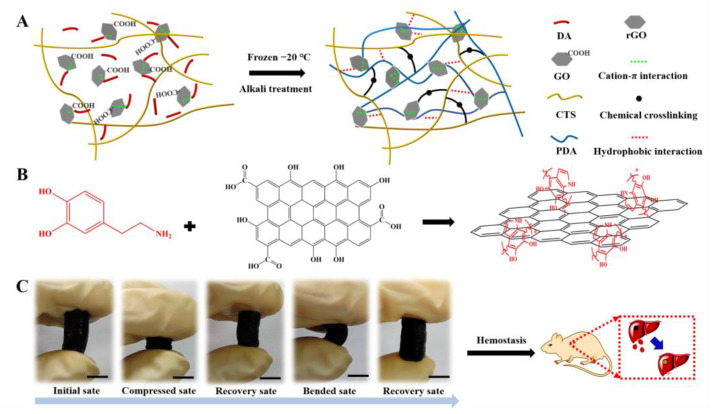
Schematic representation of the rGO-CTS cryogel. (**A**) Preparation of the rGO-CTS cryogel; (**B**) synthesis of the rGO; (**C**) photographs of the compression and bending resistance capability of the rGO-CTS cryogel: initial state, compressed state by squeezing out the free water, recovery state by absorbing water, bending and squeezing out part of the free water, and recovery state after absorbing water.

In this work, shape-memory-reduced graphene/chitosan (rGO-CTS) cryogels based on frozen-alkali treatment were prepared using a one-step method. In the one-step method, chitosan, citric acid, dopamine, and GO were evenly mixed, frozen, and soaked in an alkaline solution, where the alkaline solution induced sodium citrate to cross-link CTS and dopamine to form PDA-reducing GO. The obtained shape-memory cryogel exhibited excellent mechanical properties, antibacterial activity, and biocompatibility. In mouse liver trauma models, it also showed good blood-clotting ability and higher blood cell adhesion. These results indicate that these cryogels have great potential as non-compressible hemorrhage hemostasis dressings and wound healing materials (Figure 1).

## 2. Results and Discussion

### 2.1. Preparation and Characteristics of the rGO-CTS Cryogel

**Figure 2:**
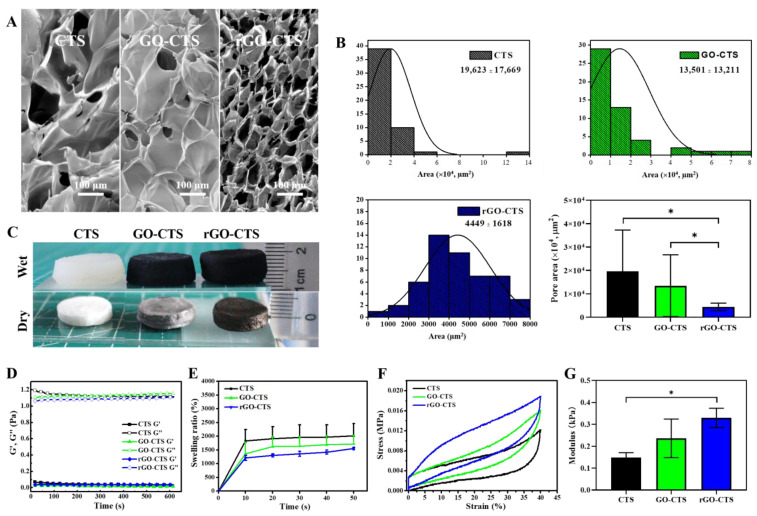
(**A**) SEM images of the CTS, GO-CTS, and rGO-CTS cryogels; (**B**) the pore area of the CTS, GO-CTS, and rGO-CTS cryogels; (**C**) electronic photographs of the CTS, GO-CTS, and rGO-CTS cryogels under dry and wet conditions; (**D**) rheological characterization of the CTS, GO-CTS, and rGO-CTS cryogels; (**E**) the swelling ratio of the CTS, GO-CTS, and rGO-CTS cryogels; (**F**) compressive stress−strain curves of the CTS, GO-CTS, and rGO-CTS cryogels; (**G**) Young’s moduli of the CTS, GO-CTS, and rGO-CTS cryogels. * *p* < 0.05.

The surface morphologies of the CTS, GO-CTS, and rGO-CTS cryogels were investigated using SEM. These cryogels had a porous network texture structure, and the rGO-CTS cryogel showed a flabbier structure with a three-dimensional network (Figure 2A). The pore areas of the GO-CTS and rGO-CTS cryogels were smaller than that of the CTS cryogel (Figure 2B). Notably, the rGO-CTS cryogel had the smallest pore area among the three cryogels. This implied that the rGO was well integrated with the matrix in the rGO-CTS cryogel because dopamine reduced GO to form rGO under alkali conditions, resulting in a dense structure.

In the liquid state, it was not gelatinous; therefore, G″ (loss modulus) was greater than G′ (storage modulus). The liquid state can be characterized by both viscosity and elasticity in rheology [11]. As shown in Figure 2D, G″ was always higher than G′, indicating that the viscosity of the solution was higher than the elasticity. There was no breakpoint in the entire process; therefore, the solution was uniform and stable.

The water uptake behavior of the cryogels is shown in Figure 2C. All cryogels contained a remarkable amount of water because of the hydrophilic nature of the main polymers. The swelling ratio of the CTS cryogel was 2012.51%, which was the highest among all cryogels and could be attributed to it having the highest pore area. The swelling ratios of GO-CTS and rGO-CTS were 1721.05% and 1554.56%, respectively (Figure 2E). Because GO plates are a physical network agent, the resistance against liquid penetration increased, and adding GO decreased the swelling behavior of the CTS cryogels. However, compared with the addition of rGO to CTS cryogels, adding GO considerably boosted the swelling behavior, owing to the existence of hydrophilic functional groups (COOH and OH). Some studies have reported that wound dressings with 100–900% capacity are the best [12]. However, the swelling ratios of the CTS, GO-CTS, and rGO-CTS cryogels reached more than 1500%, showing excellent water uptake behavior. 

As shown in Figure 2F, the strain of the three cryogels could still be restored to the initial state after cyclic compressive tests, which indicated that all cryogels had good compression elasticity and stable cryogel networks. Interestingly, the compression performance of the rGO-CTS cryogel was superior to those of the CTS and GO-CTS cryogels. The Young’s moduli of the CTS, GO-CTS, and rGO-CTS cryogels were determined to further analyze the mechanical properties of the cryogels (Figure 2G). Compared with the CTS cryogel, the Young’s moduli of the GO-CTS and rGO-CTS cryogels showed 1.59- and 2.22-fold increases, respectively. These results suggest that the introduction of dopamine-reduced graphene oxide (pGO) could improve the mechanical properties of the CTS cryogel. Moreover, the rGO-CTS cryogel did not exert severe pressure or additional damage to the wound during application.

### 2.2. Water-Triggered Shape Memory of the rGO-CTS Cryogel

**Figure 3:**
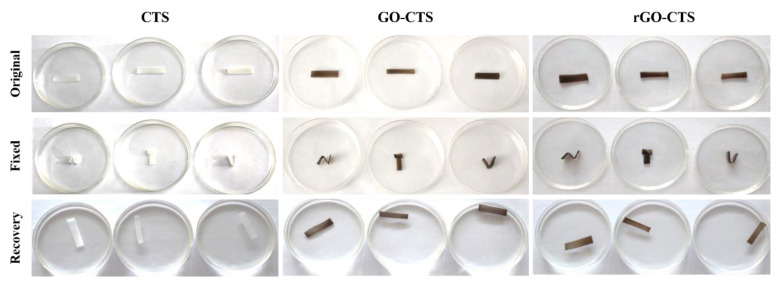
Shape-memory properties of the CTS, GO-CTS, and rGO-CTS cryogels.

Shape-memory hemostatic agents could show unique properties for hemostasis applications; they can be delivered to the wound site in the shape-fixed state by a squeeze method and recover their shape to fill the wound boundaries upon contact with bleeding blood in the human body, especially in narrow or penetrating wounds [13]. The shape-fixed state of the CTS, GO-CTS, and rGO-CTS cryogels was easily obtained by squeezing and absorbing water. The cryogels immediately recovered to their original state in less than 4 s by absorbing water after they were compressed to 40% (Video S1). The cryogels were cut into the same rectangles and then folded into complex shapes, such as N, T, and U shapes. By absorbing water, they were able to recover their state in less than 50 s (Figure 3). The shape fixity rate (R_Sf_) and shape recovery ratio (R_Sr_) of the rGO-CTS cryogel were both 100%. Whether it was a simple recovery or a more complex recovery, the recovery time of cryogels was very short and shorter than that of the chitosan/graphene oxide-based multifunctional pH-responsive hydrogel [14]. These results illustrate that the cryogels have excellent water-triggered shape-memory performance and a high recovery speed. The shape-memory capability was attributed to the reversible collapse of the pores in the cryogel matrix. The microporous structure allowed water to flow out/in freely, which possessed high polarity to absorb water and good resilience for shape recovery [15]. When the cryogels were compressed, water from the interconnected pores flowed out, and the cryogel structure collapsed. After removing the compressive stress, the stored elastic energy was released, and the cryogels exhibited a shape-fixed state. Then, the shape-fixed cryogels reabsorbed water and immediately recovered their shape and structure [4]. Therefore, the rGO-CTS cryogel possessed excellent water-triggered shape-memory capability and presented huge potential as a hemostatic agent for hemostasis applications.

### 2.3. In Vitro Blood Clotting Performance and Hemocompatibility of the rGO-CTS Cryogel

**Figure 4:**
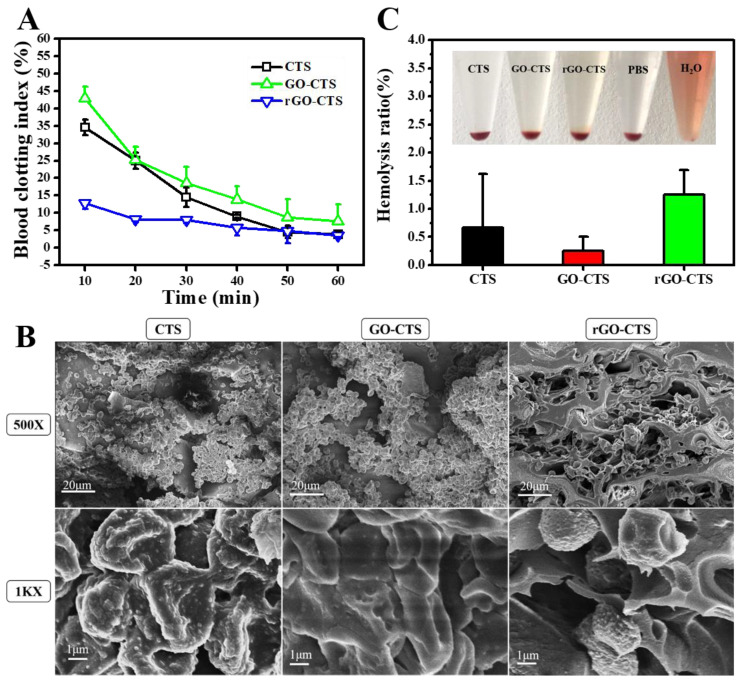
In vitro hemostatic capacity evaluation and hemolytic activity of the CTS, GO-CTS, and rGO-CTS cryogels. (**A**) In vitro dynamic whole-blood clotting evaluation of the CTS, GO-CTS, and rGO-CTS cryogels (*p* < 0.05); (**B**) SEM images of hemocyte adhesion on the CTS, GO-CTS, and rGO-CTS cryogels; (**C**) photographs from the hemolytic activity assay of the CTS, GO-CTS, and rGO-CTS cryogels using PBS as the negative control and H_2_O as the positive control.

The higher the absorbance value of the hemoglobin solution, the slower the coagulation speed. According to the above theory, the blood clotting performance of cryogels was assessed using a dynamic whole-blood clotting test [4,13]. As shown in Figure 4A, the rGO-CTS cryogel presented a lower blood-clotting index (BCI) than the CTS and GO-CTS cryogels at each time point (*p* < 0.05). These results illustrate that the three cryogels had effective blood-clotting capability, and the introduction of rGO further enhanced blooding-clotting capability. To investigate the hemostatic mechanism of the cryogels, SEM was employed to observe the surface adhesion and morphology of the blood cells on these cryogels (Figure 4B). It was found that many blood cells adhered to the three cryogels and the blood cells showed irregularly formed aggregates. Electrostatic interactions between positively charged CTS and negatively charged erythrocyte membranes cause erythrocyte agglutination and hemostasis through tissue adhesion to seal the wound [16]. Owing to the interconnected porous structure and good expansion ratio of these cryogels, they can absorb considerable amounts of blood through rapid blood absorption and blood concentration and can enhance the absorption capability of blood cells [17]. Compared to the CTS and GO-CTS cryogels, rGO-CTS had a more porous structure. Therefore, the rGO-CTS cryogel had relatively good clotting ability and great potential as a cryogel hemostatic agent in hemostatic applications.

In vitro hemolysis assays are widely employed to assess the hemocompatibility of cryogels. As shown in Figure 4C, the blood liquids of the three cryogels were centrifuged to obtain the supernatant, phosphate-buffered saline (PBS), and H_2_O as the control groups. The three cryogels were light yellow, similar to PBS, while H_2_O was bright red. According to the standard ASTM F756-17 [18], the hemolysis rates of the three cryogels, which are safe non-hemolytic materials, were less than 2%. These results confirm that the rGO-CTS cryogel possesses good hemocompatibility as a hemostatic agent and wound dressing.

### 2.4. Antibacterial Activity of the rGO-CTS Cryogel

**Figure 5:**
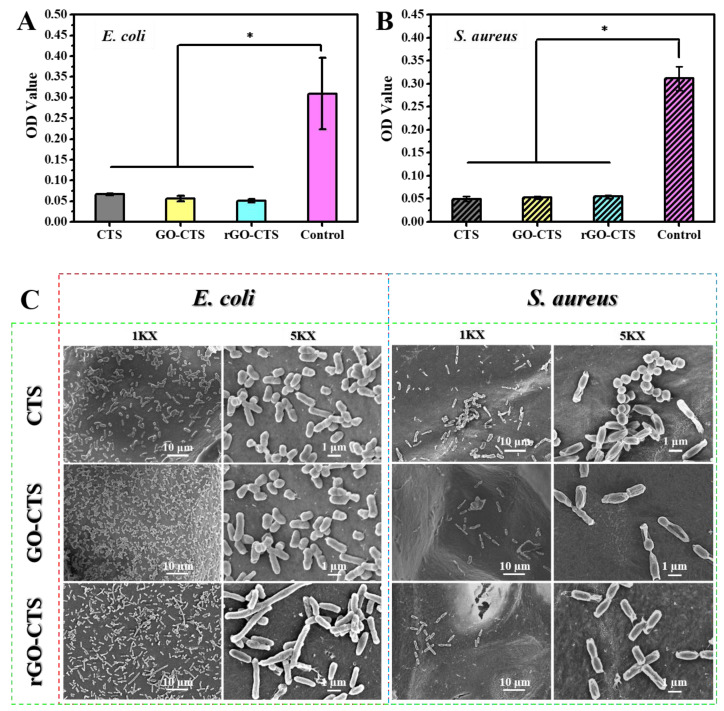
Antibacterial activity of the CTS, GO-CTS, and rGO-CTS cryogels. OD values of *E. coli* (**A**) and *S. aureus* (**B**) on the CTS, GO-CTS, and rGO-CTS cryogels, respectively; (**C**) SEM images of *E. coli* and *S. aureus* on the CTS, GO-CTS, and rGO-CTS cryogels, respectively. The SEM of *E. coli* was represented by red dotted areas, the SEM of *S. aureus* was represented by blue dotted areas, and the SEM of three cryogels was represented by green dotted areas. * *p* < 0.05.

In recent years, the antibacterial activities of CTS and its derivatives have attracted extensive attention. Wound dressings exhibit antibacterial properties. The CTS, GO-CTS, and rGO-CTS cryogels showed antibacterial activity against *Escherichia coli* (*E. coli*) and *Staphylococcus aureus* (*S. aureus*), representing Gram-negative and Gram-positive bacteria, respectively (Figure 5A,B). The OD values of the three cryogels against *E. coli* and *S. aureus* were approximately 0.05, demonstrating that the cryogels had the same inhibitory effect on *E. coli* and *S. aureus*. SEM was used to examine the morphology of *E. coli* and *S. aureus* on the three cryogels to further evaluate their antibacterial activity (Figure 5C). After incubation for 12 h, the *E. coli* was longer than the normal size, and the *S. aureus* changed from its original spherical shape to a long strip shape similar to that of *E. coli*. We hypothesized that this may be due to the strong electrostatic interaction between the rGO functional group and the bacterial membrane, which has an affinity effect. In addition, the sharp edges of rGO can rupture the bacterial membrane [19]. These results indicate that the cryogels could destroy the structure of *E. coli* and *S. aureus* to inhibit their growth and possess excellent antibacterial activity.

### 2.5. Cytocompatibility of the rGO-CTS Cryogel

**Figure 6:**
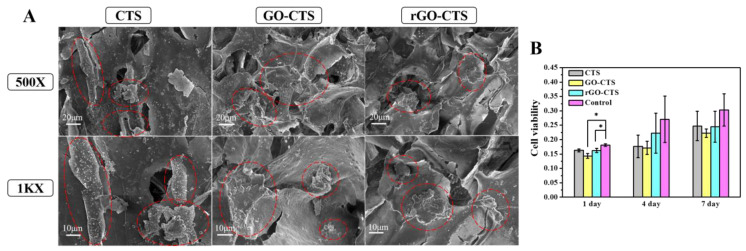
Biological assays of the CTS, GO-CTS, and rGO-CTS cryogels. (**A**) The SEM images of cells attached on the surface of the CTS, GO-CTS, and rGO-CTS cryogels, the cell clusters are represented by red dotted areas; (**B**) cell viability on the CTS, GO-CTS, and rGO-CTS cryogels. Data are presented as mean ± SD (*n* = 3), * *p* < 0.05.

Biological assays, such as cell morphology and viability of the cryogels for 7 days, were performed to confirm the excellent biocompatibility of CTS, GO-CTS, and rGO-CTS cryogels. As shown in Figure 6A, the SEM images showed cells attached to the surface of the cryogels. It was found that the cells adhered and diffused well to the cryogels, which led to the filling of the pores and reduction in the space of the cryogels. To evaluate the cell viability of the three cryogels, a cell counting kit-8 (CCK-8) test was conducted after one week of application of the cryogels (Figure 6B). Compared with the control group, the cell viability of the GO-CTS and rGO-CTS cryogels decreased after 1 d owing to the introduction of GO. However, there was no significant difference in the proliferation cells in contact with the three cryogels and the control group on days 4 and 7, indicating that the three cryogels had no obvious cytotoxicity. Therefore, the rGO-CTS cryogel exhibited good biocompatibility and could be used as a potential wound hemostatic agent or wound dressing.

### 2.6. In Vivo Hemostatic Capacity of the rGO-CTS Cryogel

**Figure 7:**
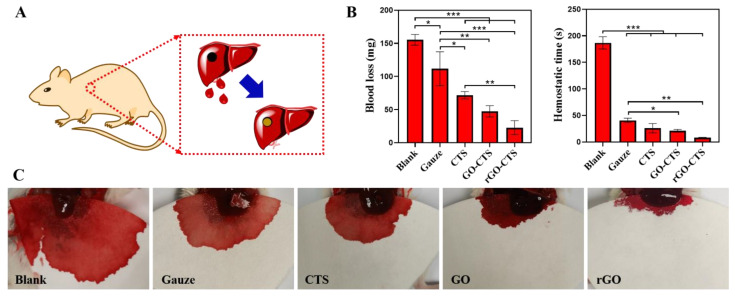
The hemostatic performance of the CTS, GO-CTS, and rGO-CTS cryogels in mouse liver trauma models. (**A**) Schematic image of the mouse liver trauma model; (**B**) blood loss and hemostatic time in the mouse liver trauma model; (**C**) photographs of in vivo hemostasis using hemostatic agents applied in cylindrical wounds of mouse liver as a non-compressible wound model, * *p* < 0.05, ** *p* < 0.01, *** *p* < 0.001.

In the mouse liver trauma model (Figure 7A), the hemostatic performance of the cryogels in vivo was further evaluated by measuring the amount of bleeding and hemostatic time. A cylindrical wound made on the mouse liver was used as the non-compressible wound model, and medical gauze was used as the control. As shown in Figure 7B,C, the blank (not-treated) group had the highest blood loss (155.33 mg) among all groups. Compared with the blank group, blood loss in all treatment groups decreased to varying degrees. The blood loss in the gauze group was the highest in the treatment group, and the blood loss in the gauze group decreased from 155.33 mg to 111.67 mg. These results indicate that medical gauze has little hemostatic effect on narrow, deep, and non-compressible bleeding. However, when CTS, GO-CTS, and rGO CTS cryogels were inserted into the bleeding site, they rapidly absorbed blood and expanded their volume to completely fill the wound site. The blood losses in the CTS, GO-CTS, and rGO-CTS cryogel groups were 71.67, 47.33, and 22.67 mg, respectively. The results regarding hemostatic time showed the same trend as in blood loss. The hemostatic time in the three cryogel groups was significantly shorter than that of the gauze, and the rGO-CTS cryogel was as short as 8.3 s. Here, we compared the hemostatic times of different hemostatic materials based on CTS. Huang et al. [20] prepared a high-strength composite cryogel hemostatic agent based on poly (vinyl alcohol) (PVA), carboxymethyl chitosan (CMCS), and dopamine (DA), which showed a hemostatic time of 2 min. The hemostasis time of the cryogel based on CNT and QCS prepared by Zhao et al. [4] was 78 s. Guo et al. prepared a QCS/PDA2.0 cryogel based on QCS and PDA with a hemostatic time of 21 s [6]. In contrast, the rGO-CTS cryogel prepared in this study had the shortest hemostatic time of 8.3 s. The CTS can interact with red blood cells and platelets and accelerate their adhesion and aggregation. CTS further enhances blood clot formation by combining almost all plasma proteins and important clotting factors to produce a series of biochemical reactions in vivo [21]. Therefore, the three cryogels exhibited good hemostatic properties for non-compressible wounds. In addition, the rGO-CTS cryogel had the smallest pore size and the highest blood-sucking ability, which can promote hemostasis. These results show that the rGO-CTS cryogel has the best hemostasis performance for non-compressible wounds and has great application potential in the hemostasis of lethal bleeding.

## 3. Materials and Methods

### 3.1. Materials

CTS (MERYER Chemical Technology, Shanghai, China), dopamine hydrochloride (Bide Pharmatech Co., Ltd., Shanghai, China), citric acid (analytically pure, Rugao Chemical Reagent Factory, Nantong, China), GO (Tanfeng Graphene Technology Co., Ltd., Suzhou, China), NaOH (analytically pure, Longxi Science Co., Ltd., Guangdong, China), acetic acid (analytically pure, Nanjing Chemical Reagent Co., Ltd., Nanjing, China), PBS (SenBeiJia, Nanjing, China), CaCl_2_ (Shanghai Zhanyun Chemical Co., Ltd., Shanghai, China), MTT assay kit (Feiyu Bio, Nantong, China), and CCK-8 (Feiyu Bio, Nantong, China).

### 3.2. Fabrication of Shape-Memory rGO-CTS Cryogels

CTS (0.21 g) was dispersed in a 3 wt% acetic acid aqueous solution, where 0.09 g citric acid was added and continuously stirred for 6 h at room temperature. Subsequently, 0.05 g of dopamine hydrochloride and 0.007 g of GO were added to the above solution and then subjected to ultrasonic treatment at a low temperature (4 °C) for 5 min. The mixture was transferred into a mold and frozen at −2 °C for 24 h. It was soaked in 0.5 mol/L NaOH solution for 1 h and completely thawed, and then washed to neutral pH with a large amount of distilled water to obtain the reduction of graphene citric acid-modified chitosan (rGO-CTS) cryogels. The CTS cryogel and GO cryogel were prepared using the above method.

### 3.3. Characterization

The prepared cryogels were freeze-dried, and their surface topography was observed using a field emission scanning electron microscope (FE-SEM, ZEISS Gemini SEM 300, ZEISS, Oberkochen, Germany). The pore area was measured using the ImageJ software, and the average pore area was calculated. The number of samples used was more than 50.

The freeze-dried cryogels were immersed in distilled water at room temperature and the equilibrated cryogels were weighed (*W_T_*). The samples were then freeze-dried and weighed again (*W_S_*). The swelling ratio (SR) was calculated as follows:

$$\mathrm{SR}\;(\%)=\frac{\left(W_{T}-W_{s}\right)}{W_{s}}\;\times \;100$$

The rheological properties of the CTS, GO, and rGO cryogels were evaluated using a Haake MARS rotational rheometer (Thermo Fisher Scientific, Waltham, MA, USA). Finally, a rheological diagram was drawn based on the obtained data.

A tensile tester was used to evaluate the compression properties of the CTS, GO-CTS, and rGO-CTS cryogels at room temperature. The cryogels were prepared in a cylindrical shape with a diameter of 15 mm and a height of 8 mm. The maximal compression strain was 40%, and the compression rate was 0.1 mm/s. After the cryogels absorbed water, their compression strain was first applied to the preset strain and then released to 0% strain. The compression-release cycle was 1 time.

### 3.4. Shape-Memory Behavior

A cylindrical cryogel with a diameter of 10 mm and height of 8 mm was compressed to 40% strain at a strain rate of 0.1 mm/s. The sample was then free of any load, the restoration process was observed, and the restoration time was recorded.

The initial shape of the cryogels was rectangular. The cryogels were fixed for 1 min using ethanol to form “N”, “T” and “U” shapes. They were then placed in a culture dish with a diameter of 70 mm, to which water was added. The cryogel shape recovery process was observed, and the recovery time was recorded. The shape fixity ratio (R_Sf_) and shape recovery ratio (R_Sr_) were determined using the following equations.

R_sf_ (%) = θ_t_/θ_0_ × 100

R_sr_ (%) = (θ_0_ − θ_f_)/θ_0_ × 100

where θ_0_ is the initial angle, θ_t_ is a fixed angle, and θ_f_ is the final angle.

### 3.5. Hemolytic Activity Assay

To evaluate the hemolysis percentage (HP) of cryogels, mouse blood was centrifuged for 10 min to obtain erythrocytes. Red blood cells (2 mL) were stabilized by sodium citrate and then diluted with PBS (2.5 mL). Cryogels (10 mg) were added to 10 mL PBS, and then a 0.2 mL erythrocyte solution was added. PBS and distilled water were used as the negative and positive control, respectively. The above solution was incubated at 37 °C for 1 h and then centrifuged at 3000 rpm for 5 min to obtain the supernatant. The absorbance of the supernatant was measured at 545 nm using a multi-labeled microplate detector (Enspire 2300, PerkinElmer, CA, USA). HP was calculated using the following formula [4]:

HP (%) = [(A_p_ − A_b_)/(A_t_ − A_b_)] × 100

where A_p_, A_b_, and A_t_ represent the absorbance values of the cryogel, negative control (PBS), and positive control (deionized water) groups, respectively. Each group consisted of three replicates.

### 3.6. Coagulation Test

First, 200 μL of CaCl_2_ (0.1 mol/L) was added to 2 mL of blood to activate the coagulation reaction. Activated blood (60 μL) was added to a 24-well plate containing the cryogels and incubated for 10, 20, 30, 40, 50, and 60 min at room temperature. The cryogel solutions were measured at 540 nm using the Enspire 2300 multi-labeled microplate detector (PerkinElemer, Waltham, WA, USA). BCI was calculated using the following formula [4]:

BCI (%) = [(*I_s_* − *I_o_*)/(*I_r_* − *I_o_*)] × 100.

*I_s_*, *I_o_*, and *I_r_* represent the absorbance values of the cryogel, negative control (PBS), and positive control (distilled water) groups, respectively. 

### 3.7. Antibacterial Activity

The cryogels were co-cultured in 24-well plates with the same concentrations of *E. coli* or *S. aureus*. After incubation for 12 h, MTT was added and the cells were incubated in the dark for 4 h. Dimethylsulfoxide was then added, and the mixture was shocked for 15 min at 37 °C. Finally, the absorbance of the bacterial solutions of the three cryogels was measured at 600 nm using the Enspire 2300 multi-labeled microplate detector (PerkinElemer).

### 3.8. Cell Cytotoxicity

After sterilizing the cryogels under UV light for 4 h, they were co-cultured with bone marrow mesenchymal stem cells (BMSCs, 1 × 10^4^ cells/well) in 24 well plates at 37 °C and 5% CO_2_ for 1, 4, and 7 days. The culture medium and the CCK-8 solution were added to the culture medium for 2 h at 37 °C, and the optical density was measured at 450 nm using the Enspire 2300 multi-labeled microplate detector (PerkinElemer). To observe the morphology of the cells in the cryogels, the cryogels were freeze-dried at −80 °C overnight and analyzed using SEM.

### 3.9. In Vivo Hemostatic Performance

The hemostatic ability of the cryogels was assessed using a mouse liver trauma model. Female mice (6 weeks old, 25–32 g) were obtained from the animal center of Nantong University and randomly and equally divided into five groups. Before hemostasis, the cryogels were cut into cylindrical shapes (Φ 3 mm) and compressed into a shrunken shape by extruding water. After anesthesia, the livers of the mice were exposed through an abdominal incision and the serous fluid around the liver was carefully removed. A cylindrical wound (Φ 3 mm) made on the mouse liver was used as the non-compressible wound model. Gauze, CTS, GO-CTS, and rGO-CTS cryogels were immediately applied to the bleeding site. Untreated wounds were used as the plank group, and each group consisted of three mice. All animal experiments were approved by the Institutional Animal Care and Use Committee of Nantong University.

### 3.10. Statistical Analysis

Experiments were conducted in triplicate, and data are presented as mean ± standard deviation (SD). Statistical significance was calculated using a one-way ANOVA with Bonferroni’s test. A *p*-value less than 0.05 was considered to indicate statistically significant differences.

## 4. Conclusions

In summary, a shape-memory rGO-CTS cryogel was fabricated based on CTS, citric acid, dopamine, and GO using a one-step frozen-alkali treatment. The alkaline solution not only promoted double cross-linking of CTS but also induced dopamine to form PDA-reducing GO. The cryogel exhibited water-induced shape-memory properties. Regardless of whether its shape was simple or complex, it could be restored to its original state in less than 50 s after immersion in water, and the fixity ratio and recovery rate were 100%. According to the results of SEM and mechanical tests, the rGO-CTS cryogel presented a uniform porous network structure and good shape recovery function. In addition, the rGO-CTS cryogel destroyed the structures of *E. coli* and *S. aureus* to inhibit their growth and possessed excellent antibacterial activity. The hemolysis rate of the rGO-CTS cryogel was less than 2% in vitro, indicating that it is a safe non-hemolytic material. In a mouse model of liver injury, the rGO-CTS cryogel rapidly absorbed blood, expanded its volume, and completely filled the wound. The hemostasis time was reduced to 8.3 s, and the blood loss was significantly lower than that in the other groups. Owing to its good clotting ability and high blood cell adhesion, the rGO-CTS cryogel can be used for wound treatment.

## Data Availability

All relevant data are presented in the manuscript; raw data are available upon request from the corresponding author.

## Supplementary Materials

The following supporting information can be downloaded at: https://www.mdpi.com/article/10.3390/ijms24021389/s1.

## References

[1] Li H., Cheng F., Wei X., Yi X., Tang S., Wang Z., Zhang Y.S., He J., Huang Y. (2021). Injectable, self-healing, antibacterial, and hemostatic N,O-carboxymethyl chitosan/oxidized chondroitin sulfate composite hydrogel for wound dressing. Mater. Sci. Eng. C Mater. Biol. Appl..

[2] Wang Y., Zhao Y., Qiao L., Zou F., Xie Y., Zheng Y., Chao Y., Yang Y., He W., Yang S. (2021). Cellulose fibers-reinforced self-expanding porous composite with multiple hemostatic efficacy and shape adaptability for uncontrollable massive hemorrhage treatment. Bioact. Mater..

[3] Yang E., Hou W., Liu K., Yang H., Wei W., Kang H., Dai H. (2022). A multifunctional chitosan hydrogel dressing for liver hemostasis and infected wound healing. Carbohydr. Polym..

[4] Zhao X., Guo B., Wu H., Liang Y., Ma P.X. (2018). Injectable antibacterial conductive nanocomposite cryogels with rapid shape recovery for noncompressible hemorrhage and wound healing. Nat. Commun..

[5] Yuan H., Chen L., Hong F.F. (2020). A Biodegradable Antibacterial nanocomposite based on oxidized bacterial nanocellulose for rapid hemostasis and wound healing. ACS Appl. Mater. Inter..

[6] Li M., Zhang Z., Liang Y., He J., Guo B. (2020). Multifunctional tissue-adhesive cryogel wound dressing for rapid nonpressing surface hemorrhage and wound repair. ACS Appl. Mater. Interfaces.

[7] Yao L., Gao H., Lin Z. (2022). A shape memory and antibacterial cryogel with rapid hemostasis for noncompressible hemorrhage and wound healing. Chem. Eng. J..

[8] Moradi S., Hamedi H., Tonelli A.E., King M.W. (2021). Chitosan/graphene oxide composite films and their biomedical and drug delivery applications: A review. Appl. Sci..

[9] Jing X., Mi H.Y., Napiwocki B.N., Peng X.F., Turng L.S. (2017). Mussel-inspired electroactive chitosan/graphene oxide composite hydrogel with rapid self-healing and recovery behavior for tissue engineering. Carbon.

[10] Chen H., Wang H., Li B., Feng B., He X., Fu W., Yuan H., Xu Z. (2018). Enhanced chondrogenic differentiation of human mesenchymal stems cells on citric acid-modified chitosan hydrogel for tracheal cartilage regeneration applications. RSC Adv..

[11] Zhou S., Han C., Ni Z., Yang C., Ni Y., Lv Y. (2022). Gelatin-oxidized nanocellulose hydrogels suitable for extrusion-based 3D bioprinting. Processes.

[12] Morgado P.I., Aguiar-Ricardo A., Correia I.J. (2015). Asymmetric membranes as ideal wound dressings: An overview on production methods, structure, properties and performance relationship. J. Membr. Sci..

[13] Fang Y., Xu Y., Wang Z., Zhou W., Yan L., Fan X., Liu H. (2020). 3D porous chitin sponge with high absorbency, rapid shape recovery, and excellent antibacterial activities for noncompressible wound. Chem. Eng. J..

[14] Nath J., Chowdhury A., Dolui S.K. (2018). Chitosan/graphene oxide-based multifunctional pH-responsive hydrogel with significant mechanical strength, self-healing property, and shape memory effect. Adv. Polym. Technol..

[15] Ying G., Jiang N., Parra C., Tang G., Zhang J., Wang H., Chen S., Huang N.P., Xie J., Zhang Y.S. (2020). Bioprinted injectable hierarchically porous gelatin methacryloyl hydrogel constructs with shape-memory properties. Adv. Funct. Mater..

[16] Keast D.H., Janmohammad A. (2021). The hemostatic and wound healing effect of chitosan following debridement of chronic ulcers. Wounds.

[17] Wang Y., Yin M., Zheng X., Li W., Ren X. (2021). Chitosan/mesoporous silica hybrid aerogel with bactericidal properties as hemostatic material. Eur. Polym. J..

[18] (2017). Standard Practice for Assessment of Hemolytic Properties of Materials.

[19] Khan M.U.A., Haider S., Raza M.A., Shah S.A., Razak S.I.A., Kadir M.R.A., Subhan F., Haider A. (2021). Smart and pH-sensitive rGO/Arabinoxylan/chitosan composite for wound dressing: In-vitro drug delivery, antibacterial activity, and biological activities. Int. J. Biol. Macromol..

[20] Huang Y., Zhao X., Wang C., Chen J., Guo B. (2021). High-strength anti-bacterial composite cryogel for lethal noncompressible hemorrhage hemostasis:synergistic physical hemostasis and chemical hemostasis. Chem. Eng. J..

[21] Hu Z., Zhang D.Y., Lu S.T., Li P.W., Li S.D. (2018). Chitosan-based composite materials for prospective hemostatic applications. Mar. Drugs.

